# Miscellaneous

**Published:** 1848-04-01

**Authors:** 


### Miscellaneous.

The Human Intellect.—The two great distinctions which mark the intellects of our species, seem to consist in the difference of character which is established by those who excel in the exercise of their perceptions and consequent recollection, and those who cultivate and discipline the energies of thought. The former are distinguished by a vigorous activity, a penetrating and unwearied observation; their curiosity seems rather to be attracted by the object itself than directed by the mind. The incessant occupation and restless inquiry furnish the memory with an abundant vocabulary: they recollect each object they have seen, and can retrace every path they have trodden; the ear greedily imbibes the conversations to which they are anxiously disposed to listen; that which they read, they verbally retain; they excel in quickness of perception and promptitude of memory, and appear to have everything by heart; they are "the gay motes that people the sunbeams" of the intellectual world:—thus we find them, as inclination may sway, accurate chronologists, biographers pregnant with anecdote, expert nomenclators, botanists, topographers, practical linguists, and bibliographers; in short, the opulent possessors of whatever perception can detect and memory preserve. The other order of men (and they are comparatively few), are the creatures of reflection: with them the senses are little on the alert; they do not fatigue the wing by excursions through the field of nature; but that which the recollection retains, becomes the subject of mental examination. An event is not registered from having merely occurred; but the causes which produced it are investigated, and a calculation is instituted concerning its probable tendency. Words are not simply regarded as the floating currency or medium of exchange, but they are severally subjected to analysis to establish their standard, or to detect the excess of their alloy; their senses are little awake to external impressions; the objects which a change of scene presents are slightly noticed and feebly remembered; their curiosity is not attracted from without, but excited from within; they are strangers to the haunts of gay and mirthful intercourse, and are rather consulted as oracles than selected as companions. This constant occupation of thought produces the philosophical historian, profound critic, physiologist, mathematician, general grammarian, etymologist, and metaphysician. After a long exertion they become disposed to melancholic disquietude, and often turn in disgust from a world, the beauties of which they want an incentive to examine, and taste to admire. Both of these intellectual orders of our species contribute, but in different manners, to the stores of knowledge. The sound, efficient, and useful mind, consists in a due balance and regular exercise of its different faculties.—*Haslam.*

Connexion of Intellectual Operations with Organic Action.—In bodily disorders, particularly in fevers, the creations of the imagination, as well as the objects of memory, are often reproduced in the eye by retransmitted impressions, which have all the brilliancy of original perceptions; and the following cases, given by Dr. Abercrombie in his

"Inquiries concerning the Intellectual Powers, and the Investigation of Truth," may be adduced in proof of this:—"A lady, whom I attended (says Dr. A.) some years ago in a slight feverish disorder, saw distinctly a party of ladies and gentlemen sitting round her bedchamber, and a servant handing something to them on a tray. The scene continued in a greater or less degree for several days, and was varied by spectacles of castles and churches, as if they had been built of finely cut crystal. The whole was in this case entirely a visual phantom, for there was no hallucination of mind; on the contrary, the patient had, from the first, a full impression that it was a morbid affection of vision connected with the fever, and amused herself and her attendants by watching and describing the changes in the scenery. The other case was that of a gentleman, who was also a patient of mine, of an irritable habit, and liable to a variety of uneasy sensations in his head. He was sitting alone in his dining-room in the twilight, the door of the room being a little open. He saw distinctly a female figure enter, wrapped in a mantle, and the face concealed by a large black bonnet. She seemed to advance a few steps towards him, and then stop. He had a full conviction that the figure was an illusion of vision, and amused himself for some time by watching it, at the same time observing that he could see through the figure, so as to perceive the lock of the door, and other objects behind it. At length, when he moved his body a little forward, it disappeared."

Intellectual Instinct of a Maniac.—In the following anecdote we recognise an extraordinary instance of that wildness and instinctive energy of intellect, so peculiar to lunatics in a state of recovery. The subject is aptly illustrative of the folly of pursuits, the expense of which is greater than the pleasure produced is worth. A physician of Milan, who undertook the cure of the insane, had a pit of water in his house, in which he kept his patients—some up to their knees, some to the girdle, and some to the chin, according to the greater or less degree of madness with which they were affected. One of the patients, who was on the point of his recovery, happening to be standing at the house-door, saw a young gentleman pass with his hawk upon fist, well mounted, and with the usual equipage of hawking, dogs, falconers, &c., behind him. The lunatic demanded to know of what use was all this preparation, and was courteously answered, to kill certain birds. "And what may be the value," said the lunatic, "of all the fowls you kill in a year?" The nobleman replied, "Five or ten crowns." "And what (rejoined the former) may your hawks, spaniels, horses, &c., stand you in within the year?" "About five thousand crowns," replied the nobleman. "Five thousand crowns!" said the lunatic; and gazing at him a moment with the wild earnestness of an approaching phrenzy, he seized him by the shoulder, and forcing him into the pit, immersed him several times in the water—(the usual practice with the physician with his most desperate patients.) Having thoroughly ducked the nobleman, he led him back to the door, and dismissed him with the following monition:—"Hark ye, my friend; take my advice, and make all possible haste from this house; for should our doctor come home, he'll drown you but what he'll cure you."

Statistics of Insanity.—The proportion in which the sexes are affected with insanity varies very much in different parts of the world. In Great Britain and Ireland, the proportion of males to females insane is stated to be as 13 to 12. In Italy, also, the number of male lunatics is greater than that of the females. But in France there are more females than males insane, in the proportion of 14 to 11. Calculating from statistical accounts derived from different parts of the globe, M. Esquirol found that the proportion of men to women insane is nearly as 37 to 38. The concurrent testimony of French and English physicians tends to show that the number of the male sex affected with lunacy, as compared with the female sex, is greater in the higher than in the lower ranks of society.

Effect of the Weather on the Mind.—It was a favourite maxim with Dr. Johnson, that the mind was independent of all external circumstances. In his conversations and periodical papers he took every opportunity of inculcating it, and indignantly scouted the idea that the weather had a necessary influence on the thoughts, &c. In his life of "Poor dear Collins," as he calls him, he ridicules very paradoxically an opinion of that unfortunate poet, that he could not write but at certain seasons. From what we know of Johnson (and of no literary man do we know more), we would suppose above all others he must have been the most sensible of the effects of our climate; and, notwithstanding Boswell's assertions to the contrary, that he was so is corroborated by his very arguments against it, into which he entered *con amore*, as though it touched him. His indigence, however, in early life, often compelled him to exertion in spite of the weather; and having thus gained some advantage over what doubtless tormented him greatly, he took revenge of it by denying its existence. Alfieri, who, in the copious memoirs of himself which he has left, made amends for his taciturnity while living, is very particular and satisfactory in the account of his *happy* moments :—"I have observed," says he, "applying to my intellect an excellent barometer, that I had greater or less genius or capacity for composition, according to the greater or less weight of the atmosphere; a total stupidity during the great solstitial and equinoctial winds; an infinitely less perspicacity in the evening than in the morning; and much more fancy, enthusiasm, and invention in mid-winter and mid-summer than in the intervening months." Alfieri gives but the facts, and leaves his readers to account for them *ad libitum*. Milton, and many others, might also be mentioned amongst those who have acknowledged themselves to be under the "skyey influences."

Moral Mania.—In a work published in the beginning of the present century, we have the following description of a class of persons who may often be found living at large, and not entirely separated from society, but who are nevertheless affected in a certain degree by this modification of insanity. There are cases in which the individuals perform most of the common duties of life with propriety, and some of them indeed with scrupulous exactness, who exhibit no strongly marked features of either temperament, no traits of superior or defective mental endowments, but yet take violent antipathies, harbour unjust suspicions, indulge strong propensities, affect singularity in dress, gait, and phraseology; are proud, conceited, and ostentatious; easily excited, and with difficulty appeased; dead to sensibility, delicacy, and refinement; obstinately riveted to the most absurd opinions; prone to controversy, and yet incapable of reasoning; always the hero of their own tale; using hyperbolic, high-flown language to express the most simple ideas, accompanied by unnatural gesticulation, inordinate action, and frequently by the most alarming expression of countenance. On some occasions they suspect sinister intentions on the most trivial grounds; on others, are a prey to fear and dread, from the most ridiculous and imaginary sources; now embracing every opportunity of exhibiting romantic courage and feats of hardihood, then indulging themselves in all manner of excesses. Persons of this description, to the casual observer, might appear actuated by a bad heart, but the experienced physician knows it is the head which is defective. They seem as if constantly affected by a greater or less degree of stimulation from intoxicating liquors, while the expression of countenance furnishes an infallible proof of mental disease. If subjected to moral restraint, or a medical regimen, they yield with reluctance to the means proposed, and generally refuse and resist, on the ground that such means are unnecessary where no disease exists; and when, by the system adopted, they are so far recovered as to be enabled to suppress the exhibition of the former peculiarities, and are again fit to be restored to society, the physician, and those friends who put them under the physician's care, are generally ever after objects of enmity and frequently of revenge.—*Cox's Practical Observations on Insanity.*

The Mental Faculties in connexion with Paralytic Affections.—Some interesting phenomena are presented by the conditions of the mental faculties connected with paralytic affections, or which remain after recovery from the apoplectic. One of the most common is a loss of the memory of words, and this has sometimes been observed to be confined to words of a particular class, as nouns, verbs, or adjectives. The patient is frequently observed to have a distinct idea of things and their relations, as well as of persons, while he is entirely unable to give them names, or to understand them when they are named to him. A singular modification of this condition has been related to me. The gentleman to whom it referred could not be made to understand the name of an object when it was spoken to him; but if the name was written down, he comprehended it immediately. Another frequent modification of the affection consists in putting one word, or one name of an object, in the place of another; and a very singular circumstance in some cases of this kind is, that the patient always applies the names in the same manner, so that those who are constantly with him come to understand exactly what he means. In one case of this kind, a gentleman, who was in other respects pretty well recovered, when he wanted coals put upon his fire, always called for paper, and when he wanted paper, he called for coals; and these names he always used in the same sense. In other cases, the patient seems to invent names, being words which, to a stranger, are quite unintelligible, but he always uses them in the same sense, and his regular attendants come to know what he means by them.

In the general pathology of paralysis, there is much obscurity. We find it connected with a great variety of morbid conditions of the brain; and on the other hand, we find all these existing without producing it. We cannot attempt to explain these difficulties, and must content ourselves with a simple view of the facts as they stand in the present state of our knowledge.—*Abercrombie on Diseases of the Brain.*

Hydrophobia.—M. Salin seems to have been the first who conjectured that in this horrible disease the spinal cord is affected; and a case is related in Dr. Johnson's "Medicò-Chirurgical Journal" for October, 1817, which seems to afford some probability to the conjecture. The case was well marked, violent, and speedily fatal. The membranes of the brain were found highly vascular, with considerable serous effusion; but the principal marks of the disease were in the coverings of the pons Varolii, medulla oblongata, and the upper part of the spinal cord. These parts are said to have formed one crust of intense inflammation, and on the spinal cord this crust was more intense than in any of the other parts.—*Ibid.*

Memory.—Among the phenomena of memory there are two very curious occurrences, and for which no adequate explanation has been hitherto afforded. Many of the transactions of our early years appear to be wholly obliterated from our recollection; they have never been presented as the subject of our thoughts, but after the lapse of many years have been accidentally revived by our being placed in the situation which originally gave them birth. Although there are numerous instances on record, and some, perhaps, familiar to every reader, I shall prefer the relation of one which came under my immediate observation. About sixteen years ago (1803) I attended a lady at some distance from town, who was in the last stage of an incurable disorder. A short time before her death, she requested that her youngest child, a girl about four years of age, might be brought to visit her, and which was accordingly complied with. The child remained with her about three days. During the last summer some circumstances led me to accompany this young lady to the same house. Of her visit when a child she retained no trace of recollection, nor was the name of the village ever known to her. When arrived at the house she had no memory of its exterior, but on entering the room where her mother had been confined, her eye anxiously traversed the apartment, and she said, "I have been here before; the prospect from the window is quite familiar to me, and I remember that in this part of the room there was a bed and a sick lady, who kissed me and wept." On minute inquiry, none of these circumstances had ever recurred to her recollection during this long interval, and in all probability they would never have recurred but for the locality which revived them. In a work, professedly the fabric of fancy, but which is evidently a portrait from nature, and most highly finished, in the third volume of "Guy Mannering," the reader may peruse a similar but more interesting relation, where the return of Bertram to the scenes of his childhood awakens a train of reminiscences which conduce to the development of his history and legitimate claims.—*Haslam on Sound Mind.*

